# Windows of Integration Hypothesis Revisited

**DOI:** 10.3389/fnhum.2020.617187

**Published:** 2021-01-14

**Authors:** Rony Hirschhorn, Ofer Kahane, Inbal Gur-Arie, Nathan Faivre, Liad Mudrik

**Affiliations:** ^1^Sagol School of Neuroscience, Tel-Aviv University, Tel-Aviv, Israel; ^2^School of Psychological Sciences, Tel-Aviv University, Tel-Aviv, Israel; ^3^Laboratoire de Psychologie et Neurocognition (LPNC), CNRS UMR 5105, Université Grenoble Alpes, Grenoble, France

**Keywords:** consciousness, unconscious processing, integration, windows of integration, visual perception, unconscious integration

## Abstract

In the ongoing research of the functions of consciousness, special emphasis has been put on integration of information: the ability to combine different signals into a coherent, unified one. Several theories of consciousness hold that this ability depends on – or at least goes hand in hand with – conscious processing. Yet some empirical findings have suggested otherwise, claiming that integration of information could take place even without awareness. Trying to reconcile this apparent contradiction, the “windows of integration” (WOI) hypothesis claims that conscious access enables signal processing over large integration windows. The hypothesis applies to integration windows defined either temporally, spatially, or semantically. In this review, we explain the hypothesis and re-examine it in light of new studies published since it was suggested. In line with the hypothesis, these studies provide compelling evidence for unconscious integration, but also demonstrate its limits with respect to time, space, and semantic distance. The review further highlights open questions that still need to be pursued to demonstrate the applicability of the WOI hypothesis as a guiding principle for understanding the depth and scope of unconscious processes.

## Introduction

What are the relations between integration and consciousness? This question has not ceased to intrigue scholars across disciplines (for review, see Mudrik et al., 2014) and is still unresolved. Integration – defined here as a process whereby individual signals are combined to a novel, unified representation – has been repeatedly suggested to be tightly related to consciousness (Dehaene and Changeux, 2011; Oizumi et al., 2014; Ruffini, 2017; Safron, 2020). Yet the nature of these relations is unclear, some studies report evidence for different types of integrative processes taking place without awareness (to be reviewed below; a few examples are the integration of 3D objects: Cho and He, 2019; integration of names and professions: Scott et al., 2018; and integration of successive arrows: Tu et al., 2020). On the other hand, others negate these findings and either report no evidence for unconscious integration (e.g., no difference between congruent and incongruent masked idioms: Zhou et al., 2016) or show that previous findings fail to replicate (e.g., Moors et al., 2016a; Rabagliati et al., 2018).

A possible explanation for these conflicting findings was put forward as part of the Windows of Integration hypothesis (WOI; Mudrik et al., 2014). According to this suggestion, the need for conscious processing in integrating two or more representations into a novel one depends on the size of the integration window, which can be defined spatially, temporally, or semantically^1^. And so, integration can occur both with and without consciousness for small windows of integration, but only consciously for larger ones. This hypothesis seemed to reconcile previous findings, as unconscious integration in those studies was indeed typically confined to small integration windows (e.g., short distances between integrated stimuli, short intervals between them, or a small semantic distance).

Yet in recent years, new studies have tackled the question of unconscious integration, allowing us to revisit the original hypothesis and see if it is borne out by the data, should be updated or completely abandoned. In this narrative review, we describe the main findings of these papers (published since 2013; for a full list and classification of papers, see Supplementary Data, and for a review of earlier effects, see Mudrik et al., 2014), and try to classify them according to window size. Note that this is not a quantitative review nor a meta-analysis; hence, we take the authors’ interpretation of their findings at face value and do not attempt to assess the statistical reliability of the effects.

## Windows of Integration

We define integration as the formation of a unified representation from distinct signals spread over time, space, or semantic distances.

*Semantic integration* refers to combining the meaning of two symbols into a new one. This ability to judge the relations between two elements and to integrate them into a new representation is sometimes referred to as relational processing (Goodwin and Johnson-Laird, 2005; Zucker and Mudrik, 2019). It is often claimed to underlie processes like word and sentence comprehension (van Gaal et al., 2014), arithmetic operations (Sklar et al., 2012), similarity judgments (Liu et al., 2016), and categorization (Ruch et al., 2016; Biderman et al., 2020). Accordingly, such integration is prominent and has been widely studied in a myriad of processes, ranging from perceptual processing (Bar, 2004) to verbal and conceptual ones (Halford et al., 2010). The Semantic Processing Integration Window (SPIW) is defined by the semantic distance between the units of integrated information, which could also be defined in terms of semantic complexity; the more complex the integration (i.e., the more semantic processing it requires), the bigger the window.

*Temporal integration* refers to the combination of sequential inputs into a unified representation (Mudrik et al., 2014; Faivre and Koch, 2014b). It allows perceiving events as unfolding in time (Faivre and Koch, 2014a,b; Salomon et al., 2016; Moors et al., 2017b), inferring the relations between successive events (Tzovara et al., 2015a; Schlossmacher et al., 2020; Tu et al., 2020), or combining them into one meaningful signal (Zhou et al., 2016; Yang et al., 2017; Nakamura et al., 2018; Mongelli et al., 2019; Tu et al., 2019b). This type of integration, too, can take place over windows of varying sizes, defined here as Temporal Integration Windows (TIWs). Operationally speaking, this is defined as “the maximal delay between two events for which a response differs from the summed responses associated with each event” (Faivre and Koch, 2014b). Note that this definition is different from other usages of “temporal integration window” in the literature; for example, some use it to refer to the brain’s temporal “resolution” (Arnett and Di Lollo, 1979; Blake and Lee, 2005; Wutz et al., 2016), a topic that has been the focus of recent clinical (Stevenson et al., 2014; Zhou et al., 2018) and developmental research (Freschl et al., 2019; Ronconi et al., 2020) and is related to hypotheses about whether consciousness is continuous or discrete (for a review, see Herzog et al., 2020).

Finally, *spatial integration* is defined across space; that is, combining information or signals located at different places in space, into one representation. This can take place throughout the visual field, both within the foveal region, which occupies about 2° in the center of the visual field and is characterized by high acuity (Curcio et al., 1987; Cohen et al., 2020), and within the parafoveal and the peripheral regions surrounding it (Rayner, 2009). Notably, we are referring here to the visual spatial area, as most studies in the field have focused on visual processing (in line with the seminal framework by Crick and Koch, 2003), yet spatial integration can also take place over the auditory, somatosensory and even olfactory space. In all cases, integration is a combination between two signals, that are spatially distinct. Accordingly, Spatial Integration Windows (SIWs) are defined based on the (physical) distance between the integrated signals. Thus, the larger the distance between elements, the larger the integration window.

**FIGURE 1:**
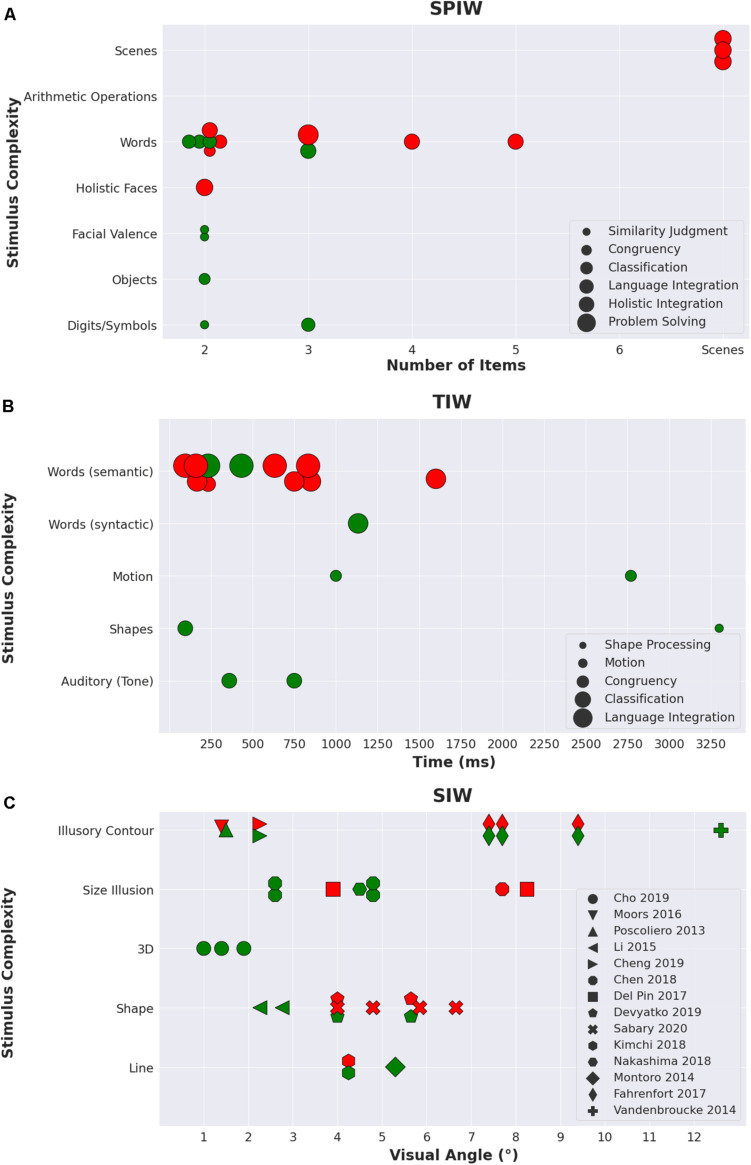
An illustration of findings of studies of unconscious integration published since 2013, outlined according to SPIW, TIW, and SIW. For all panels, red markers indicate experiments that were interpreted by the authors as suggesting no integration over that window, and green markers represent experiments that were interpreted by the authors as showing positive evidence for unconscious integration. **(A)** SPIW: the *x* axis denotes the number of integrated items, and the *y* axis describes the type of stimuli in the experiment, ordered in ascending complexity, as interpreted by the authors. Marker size describes the level of cognitive complexity the task required, as interpreted by the authors (in ascending order: similarity judgment, congruency, classification, language integration, holistic integration, and problem solving. In the absence of a clear metric of complexity, the suggested order is based on our assessment of the required cognitive effort). **(B)** TIW: the *x* axis denotes the duration of the integration window, and the *y* axis describes the type of stimuli in the experiment, ordered in ascending complexity, as interpreted by the authors. Marker size describes the level of cognitive complexity of the task, as interpreted by the authors (in ascending order: shape processing, motion inference, congruency, classification, language integration). **(C)** SIW: the *x* axis denotes the visual angle of the area captured by the integrated items, and the *y* axis describes the complexity of the visual stimulus, as interpreted by the authors (in increasing order: line, shape, 3D, size illusion, and illusory contour). Experiments that belong to the same research paper are marked with an identical shape.

For all integration types, the WOI hypothesis can be broken down to two parts. The first holds that unconscious integration can take place for small integration windows (henceforth, *WOIH1*). The second states that this will not be the case for large windows; there, conscious processing would be needed for integration to occur (*WOIH2*). Inspecting the literature, no clear-cut answer emerges with respect to these premises across types of integration windows (Figure 1), especially since very few studies put the hypotheses to a direct test, by manipulating window size within one study. Still, when the mutual influences between window types are taken into account (for example, when semantic differences are considered with respect to studies that probed temporal integration) and when the studies are grouped together under the WOI framework, the main principle does seem to be confirmed, especially for the semantic domain, which constitutes the lion’s share of the data.

### Semantic Processing Integration Window

Words are the most common stimulus used in unconscious semantic integration studies (e.g., Zabelina et al., 2013; van Gaal et al., 2014; Scott et al., 2018; Mongelli et al., 2019). Many studies found evidence supporting WOIH1, reporting different forms of unconscious integration between two and sometimes even three words, mostly at the pre-sentence level, for tasks that seem to require relatively shallow processing; behaviorally, response-time congruency effects were evoked by categorically related pairs of words, compared to unrelated pairs (e.g., Pear and Orange vs. Pear and Hammer in Tu et al., 2019a). Similarly, a two-item integration was also found between words and non-words, and between words and pictorial stimuli (Ruch et al., 2016). Surprisingly, this effect was reported to last for 15–25 min, as opposed to typical findings in the field that are short-lived (Greenwald et al., 1996). Another study reported integration between three masked Chinese words that either form or do not form an idiom with a sequentially presented visible target (Tu et al., 2019b; see further discussion in the TIW section). However, note that the latter finding could simply rely on familiarity or well-established templates for these idioms, rather than on semantic integration between the words to form a sentence-level understanding. Using EEG, unconscious integration between two simultaneously presented words [a modifier (very/not) and an adjective] was reported, when a similar N400 effect (held by some to index semantic integration; Kutas and Federmeier, 2011) was found for masked and unmasked words (van Gaal et al., 2014). And with fMRI, differences were found between meaningful sentences and non-words rendered unconscious (Axelrod et al., 2015), though it is more likely that this represents differential processing between words and non-words, irrespective of integration (accordingly, it was not included in the figure).

Unconscious two-item associations are not restricted to the visual domain; in one study, the relations between pairs of unconsciously presented visual and auditory digits were processed, but only following conscious training (Faivre et al., 2014). A more recent study found that subliminal associations can be formed between two visually presented words, auditorily presented ones, and visual-auditory ones even without prior conscious training (Scott et al., 2018). Taken together with the abovementioned visual studies, these findings support WOIH1, suggesting that some forms of semantic integration, mostly involving two and sometimes three items at the pre-sentence level, are indeed possible even without awareness.

The data tells a more complicated story when it comes to WOIH2. Here, the ultimate test lies in studies that include some manipulation of window size, so to directly probe the hypothesis within one experiment. Unfortunately, such studies are relatively rare (though there are more in the spatial domain; see the SIW section below). One study did inspect subjects’ ability to integrate an unconsciously perceived contextual cue and a visible ambiguous stimulus (Biderman et al., 2020). There, unconsciously perceived numbers (12 and 14) or letters (A and C) biased the classification of the visible ambiguous stimulus that could be interpreted as B or 13. Interestingly, similar facilitation was not found when the invisible contextual inducers formed a word with the visible ambiguous stimulus, suggesting that unconscious integration was limited to the categorical level and did not occur at the lexical one, in line with WOIH2.

Yet another way to assess this hypothesis is to examine studies that did *not* find evidence for unconscious integration, and contrast them with studies that did: do they differ in the size of probed SPIW? Overall, the answer seems to be positive, at least under the interpretation we suggest here. First, integration that involves more complicated, deeper processing (i.e., greater semantic complexity which can be considered as a larger SPIW), is less likely to occur unconsciously, much like the conclusion of the B/13 study above. For example, an ERP study failed to find differences between waveforms evoked by masked word-pairs that either correctly or incorrectly described a subsequent visible target picture (Mongelli et al., 2019). There, the task involved integrating the meaning of two independent words into a short sentence with a subject and verb, as opposed to the studies reviewed above that probed similarity judgments (Tu et al., 2019a) or the integration of a word and a modifier (van Gaal et al., 2014). Sentence-level reading was put into question in another recent study (Rabagliati et al., 2018) that failed to replicate an earlier finding (Sklar et al., 2012) of unconscious integration of four-to-five-word sentences, or even two-word phrases (note that these studies inevitably also manipulate either spatial distance or temporal one, depending on whether the stimuli are presented simultaneously or sequentially). An interesting example is a study that presented problems (or riddles) where three seemingly unrelated words (e.g., pine, crab, and sauce) were related to a fourth word (here, apple), without being related to each other (Zabelina et al., 2013). These riddles were preceded by either the same three words or different ones, rendered invisible. Subjects were better at solving these problems following the presentation of the same words, compared with different ones, which seemingly suggests relatively deep semantic integration. Intriguingly, however, the effect was only found when subjects indicated that they solved the problem analytically, rather than based on insight. The authors argued that the lack of effect across all trial types indicates that semantic integration actually did not take place; if it had, they explain, it should have been observed also in the insight condition. Instead, they claim that suppressed words evoked enough semantic priming to activate associations that are related to correct solutions, even without integration (e.g., automatic spread of activation). If we accept their claim for lack of integration, this supports WOIH2, as this study requires the integration of relatively large number of items (as most findings support unconscious integration within a two-item SPIW size, while a three-item SPIW is less prevalent), and – more importantly – a much deeper semantic processing than the one required in the studies where effects were found.

Moving to non-verbal stimuli, unconscious integration was implied for simple stimuli comprised of object-pairs; the latter broke suppression faster when they were congruently positioned (i.e., mirror above sink) than incongruently positioned (i.e., sink above mirror; Stein et al., 2015). Yet object-scene relations, which require higher-level of integration, have been recently shown to require conscious processing, as opposed to earlier reports (Mudrik et al., 2011; Mudrik and Koch, 2013); this was demonstrated both behaviorally (Moors et al., 2016a; Biderman and Mudrik, 2018) and using fMRI (Faivre et al., 2019). Finally, in face processing, congruency effects of valence-relations in subliminal pairs of faces were found both at the neural (Tu et al., 2013) and behavioral (Liu et al., 2016) levels. However, when contextual effects requiring holistic processing of faces were probed, no effects were found (Axelrod and Rees, 2014). Though it might be claimed that holistic processing is deeper than valence similarity judgment, this claim is highly speculative and requires further research.

Taken together, the above studies seem to support the WOI hypothesis, showing first that semantic integration can occur within small SPIWs, and second that this capacity declines and eventually diminishes as complexity of information and depth of processing increases.

### Temporal Integration Window

As for temporal integration, there are two types of studies that probed conscious and unconscious integration over time. One line of research did so by suppressing moving stimuli and checking if motion was processed. There, some studies found evidence for motion processing that lasts as long as a few seconds (Salomon et al., 2016; Moors et al., 2017b). Notably, however, deciphering the exact TIWs in such studies is somewhat problematic, as we cannot know for sure which portion of the movement was processed (i.e., what was the actual TIW). To explain, even if the presented motion lasts for a whole second, it might be that processing takes place over the last/first tens of milliseconds (though not for all papers; See Salomon et al., 2016 for an example for such exception), not integrating over the entire period (for a discussion of motion as part of the continuous vs. discrete consciousness debate, see again Herzog et al., 2020). Thus, it might be more informative to focus on the second line of research, where two (or more) distinct stimuli, spaced in time, must be integrated to elicit differential responses. There, the only way to process the relations between the two stimuli, or to combine them into a new meaning, would be to integrate over the time window between them.

Much like in the semantic integration section, only one study parametrically manipulated the temporal distance between stimuli, using EEG to find evidence for integration of words presented at different inter-stimulus intervals (Nakamura et al., 2018). Reminiscent of Faivre and Koch (2014b), the paper reports unconscious integration of words in a sentence over small TIWs, but not large ones (notably, this effect was only found in EEG and not behaviorally). This again supports both WOIH1 and WOIH2, though the effect might also be explained by semantic complexity, since in this study, longer TIWs translated into more words. Additional support for WOIH2 can be found in studies using series of auditory stimuli with both local and global deviations. These studies typically find local unconscious integration (Faugeras et al., 2012; Tzovara et al., 2015a), while global integration – spanning over larger windows and notably more stimuli – is under debate (cf. Naccache et al., 2015; Tzovara et al., 2015b), in line with WOIH2. On the other hand, two papers found integration over much larger windows (Hung and Hsieh, 2020, TIW: 1,333 ms; Schlossmacher et al., 2020, TIW: 3,300 ms), which stands in sharp contradiction to the findings by Nakamura and colleagues reported above. How can these discrepancies be reconciled within one framework?

A possible solution lies in the type of integration probed in each of these studies, or the interplay between temporal and semantic integration windows. In Hung and Hsieh’s study, where unconscious integration over a large TIW was found, the integration was syntactic, while in the Nakamura et al. paper, it was semantic. In the study by Schlossmacher and colleagues, a series of visual stimuli was presented, and EEG indicated deviation processing, so again – high-level semantic integration was not required. Presumably, there might be a dependency between temporal and semantic integration windows, so that for lower-level integration (semantic-wise), unconscious processes can take place over larger temporal windows (Hung and Hsieh, 2020). For higher-level semantic relations, conversely, unconscious integration might either diminish with respect to TIW size (Nakamura et al., 2018), or be needed even for smaller TIWs (van Gaal et al., 2014; Zhou et al., 2016; Yang et al., 2017; Mongelli et al., 2019; Tu et al., 2019b). Thus, it could be that the temporal domain is secondary to the semantic one. Indeed, for the same TIW (96 ms) and paradigm (masking), one study found unconscious integration of the relation between two arrows (Tu et al., 2020), while another study failed to find unconscious integration of two words (Tu et al., 2019b). This hints that the temporal window over which unconscious integration can take place might differ with the degrees of semantic relations. Notably, in the absence of studies that attempted to find high-level semantic effects over large temporal windows, the picture is partial, so at this point, this suggestion is only based on anecdotal findings, and more research is needed to determine if the WOI hypothesis is correct with respect to the temporal domain. Such research should systematically manipulate both temporal and semantic windows, to disentangle the effects each of them has on the prospects of integration occurring without awareness.

### Spatial Integration Window

Unlike the semantic and temporal windows, SIW-size manipulations (accomplished by changing the area captured by the stimulus or using stimuli of different sizes, measured by visual angle) are more common in unconscious processing studies (Fahrenfort et al., 2017; Chen et al., 2018; Cho and He, 2019; Sabary et al., 2020). Yet these manipulations did not seem to evoke differential patterns within each study (i.e., either an effect was found for all distances, or it was not found for any of them), perhaps since the different SIWs were relatively close to each other [e.g., 1.9 vs. 1.4° in one study (Cho and He, 2019), or 4, 4.8, 5.85, and 6.66° in another (Sabary et al., 2020)]. Collapsing all these studies together, while taking into account other studies that only probed one SIW, suggests that unconscious integration can generally take place for windows smaller than 3° (Chen et al., 2018, SIW: 2.6°; Cheng et al., 2019, SIW: 2.26°; Cho and He, 2019, SIW: 1.0, 1.4, and 1.9°; Li and Li, 2015, SIW: 2.26 and 2.76°; and Poscoliero et al., 2013, SIW: 1.5°), thus supporting WOIH1. For larger windows, the results are less coherent; some studies point to a lack of unconscious integration for windows larger than 4° (Chen et al., 2018, SIW: 7.7°; Del Pin et al., 2017, SIW: 8.25°; Devyatko et al., 2019, SIW: 4.0°, 5.65°; Kimchi et al., 2018, SIW: 4.25°; Sabary et al., 2020, SIW: 4.0, 4.8, 5.85, and 6.66°), while others find integration in similar and even larger windows (Chen et al., 2018, SIW: 4.8°; Devyatko et al., 2019, SIW: 4.0° and 5.65°; Kimchi et al., 2018, SIW: 4.25°; Montoro et al., 2014, SIW: 5.3°; Nakashima and Sugita, 2018, SIW: 4.5°; and Vandenbroucke et al., 2014, SIW: 12.6°). Note that in all these studies, two or more stimuli presented centrally had to be integrated to form a coherent representation, and window size refers to the area over which this integration took place [an exception is the study by Cho and He (2019), where a 3D cube was presented, and had to be integrated to yield an orientation-adaptation effect; we included it here as it was indeed interpreted by the authors as a case of structural organization and integration].

How can this discrepancy in findings for unconscious spatial integration over medium and large-sized windows be explained? One possible explanation relates to the method used for rendering stimuli unconscious. Attention-based paradigms, such as inattentional blindness (IB) and attentional blink (AB), seem to allow unconscious spatial integration over surprisingly large SIWs (Fahrenfort et al., 2017, AB, SIW: 7.4, 7.7, and 9.4°; Vandenbroucke et al., 2014, IB, SIW: 12.6°), that was not found using masking (Fahrenfort et al., 2017). This pattern was also demonstrated when evidence for unconscious integration of a stimulus using IB (Pitts et al., 2012) was not replicated with masking (Pitts et al., 2014). Interestingly though, studies directly comparing between Continuous Flash Suppression (CFS; Tsuchiya and Koch, 2005) and masking reported unconscious integration for masked stimuli, but no indication for integration of the same stimuli under CFS (Kimchi et al., 2018; Devyatko et al., 2019). Even with small SIWs, in studies of unconscious integration of a Kanizsa configuration (Kanizsa, 1976), integration was reported when using masking (Poscoliero et al., 2013, SIW: 1.5°) but not under CFS (Moors et al., 2016b, SIW: 1.4°). These findings are consistent with the hypothesis that stimulus integration under CFS is limited (Moors et al., 2017a; but see Sklar et al., 2018). Thus, it seems like attentional paradigms allow more unconscious spatial integration than CFS, and conflicting findings are found for masking (for more discussion about the difference between paradigms, see Izatt et al., 2014; Breitmeyer, 2015).

And so, both stimulus complexity and the method by which stimuli are rendered unconscious impact unconscious integration, beyond the size of the SIW as defined by visual angles. Similar to TIWs, then, the complexities of the data require further empirical examination, while controlling for the above factors.

## Concluding Remarks

Taken together, the studies reviewed here provide suggestive though insufficient evidence for the WOI hypothesis; its first claim – WOIH1 – does seem to be confirmed by the data, as multiple studies report integration of information without awareness. Yet what is still suggestive at this point, is that this effect depends on window size; there is evidence in that direction, especially when examining SPIWs, which were more widely studied than TIWs or SIWs: semantic integration does seem to depend on the number of integrated items as well as the complexity of the semantic relations between them. A more complicated, yet somewhat similar, image emerges for TIWs and SIWs. There, for the most part, studies do follow WOIH2, showing that unconscious integration is more likely to be found for smaller temporal/spatial windows. However, some studies either fail to find integration for small windows or find integration for large ones. The picture becomes clearer when the findings are inspected through a multi-dimensional prism, also taking semantic complexity into account. In both the temporal and spatial domains, semantic relations and/or complexity seem to be critical in determining whether integration can take place unconsciously or not. Especially for the temporal domain, there is not enough data to inspect the effect of increasing the TIW within the same level of semantic complexity. This further strengthens the need for dedicated studies that would test the WOI hypothesis directly, by manipulating one type of window while keeping other windows constant. Currently, there are simply not enough studies that test the effect of window size directly, without confounding factors.

In sum, the WOI hypothesis aims at delineating the borders between integration processes that require conscious perception and those that can occur without awareness. This guiding principle seems to be supported to some extent by recent findings in consciousness studies; however, more data is needed to provide a definitive answer on the matter, especially with respect to the temporal and spatial domains. Generally speaking, this review also highlights the often-overlooked complexities of studying unconscious processing, which might explain conflicting findings in the field. Unconscious processes depend not only on the manipulation of interest, but on many additional factors that might affect the results, much like we saw here with both the temporal and spatial windows studies. In our field, the devil really is in the details (see also Rothkirch and Hesselmann, 2017), so future studies should take that into account when designing their experiments, to make sure the critical feature of interest is isolated, with minimal influences from other domains.

## Author Contributions

RH, IG-A, and OK searched the literature, reviewed all relevant manuscripts, and discussed them with LM. RH focused on temporal integration windows, OK on semantic integration windows, and IG-A on spatial. RH combined these sections and wrote the manuscript, prepared the figure and the Supplementary Table. NF and LM reviewed and edited the manuscript. All authors contributed to the article and approved the submitted version.

## Conflict of Interest

The authors declare that the research was conducted in the absence of any commercial or financial relationships that could be construed as a potential conflict of interest.

## Footnotes

1 The original hypothesis also referred to multi-modal integration windows, defined by the number of integrated modalities. Since this has been seldomly studied, multi-modal windows are not included in this review.
